# ﻿Solved at last: The Philippine endemic *Psychotriaphilippensis* is a synonym of *Scyphiphorahydrophylacea* (Rubiaceae, Scyphiphoreae)

**DOI:** 10.3897/phytokeys.221.97766

**Published:** 2023-03-10

**Authors:** Andreas Berger

**Affiliations:** 1 Department of Botany and Biodiversity Research, University of Vienna, Rennweg 14, A-1030, Vienna, Austria University of Vienna Vienna Austria; 2 Department of Botany, Natural History Museum Vienna, Burgring 7, A-1010, Vienna, Austria University of Vienna Vienna Austria

**Keywords:** Chamisso, herbarium history, Paleotropical Rubiaceae, The Philippines

## Abstract

*Psychotriaphilippensis* (Rubiaceae) was described by Chamisso and Schlechtendal in 1829, was the first *Psychotria* name published for the Philippines and is currently considered a Philippine endemic. The name remained in a taxonomic limbo for almost two centuries as it was variously accepted, synonymized or considered obscure, probably because the type specimen in the Berlin herbarium was destroyed and no original material has survived or is currently known. A combined analysis of the information on morphology, type locality and ecology contained in the protologue and a review of relevant literature on the study of the name by various authors over the last two centuries finally clarified the identity of *P.philippensis*. The name is confirmed here as a synonym of the rubiaceous mangrove *Scyphiphorahydrophylacea*, as first proposed by Schumann, one of the authorities of the family in the late 19^th^ century, and the application of *P.philippensis* is fixed by neotypification. This reduces the number of Philippine species of *Psychotria* by one, but fortunately, this is not happening through extinction, as has been the case with too many species of the highly endangered Philippine flora. In addition, the history of the discovery and study of *S.hydrophylacea* and its synonyms are described in detail, and one lectotype and one neotype are designated.

## ﻿Introduction

The Philippines is one of the world’s top 25 biodiversity hotspots and one of the eight “hottest hotspots” in terms of endemic species and habitat loss (Myers et al. 2000; Brooks et al. 2006; Posa et al. 2008; Webb et al. 2010). One of the largest groups in the Philippine flora and the tropics is the coffee family (Rubiaceae), with more than 13,000 described species. Rubiaceae is represented in the Philippines by more than 455 native species in 74 genera and 25 tribes (Alejandro 2007). Merrill (1923–1925, 1923a, b, 1926) published the first complete account and enumeration of Philippine flowering plants and formed the basis for subsequent research on the Philippine Rubiaceae. Later, Alejandro and Liede (2003) and Alejandro (2007) updated Merrill's list with species described since then and changes in generic concepts, but noted that some groups are still poorly known due to the size and taxonomic complexity of the family and the need for modern revision. Fortunately, several recent monographic studies have greatly improved our knowledge of the family in the region (e.g., Alejandro et al. 2010, 2011, 2016; Wong and Low 2011; Obico and Alejandro 2012; Pereira and Ridsdale 2012; Chavez et al. 2020).

Among the genera revised recently in the Philippines, is *Psychotria* (Psychotrieae), the largest and most complex genus of Rubiaceae in the Philippines and elsewhere. Sohmer and Davis (2007) recognized 112 *Psychotria* species and 17 infraspecific taxa, of which 106 are endemic and 25 are only known from the type. The broad circumscription of *Psychotria* was subsequently narrowed following morphological and phylogenetic studies, which led to the exclusion of numerous segregates in the tribe Palicoureeae (Nepokroeff et al. 1999; Robbrecht and Manen 2006; Razafimandimbison et al. 2014). On the other hand, long-recognized segregates such as the myrmecophytic Hydnophytinae C.R.Huxley & Jebb (or the genera *Cremocarpon* Boivin & Baill. and *Pyragra* Bremek. not present in the Philippines) were found to be nested in *Psychotria*. In the current delimitation of *Psychotria*, its center of diversity lies in the Palaeotropics, and it includes at least 1,600 species of trees, treelets, shrubs, subshrubs, herbs, climbers and epiphytes (Razafimandimbison et al. 2014). Following these authors, the two species comprising the Membranifolia Species Group in Sohmer and Davis (2007) were excluded from *Psychotria* and transferred to an expanded *Eumachia* within Palicoureeae (Taylor et al. 2017).

With few exceptions, all of the 110 remaining *Psychotria* species are probably endemic to the Philippines, but a full assessment would require a better knowledge of the Southeast Asian and especially the Bornean species. They are predominantly found in primary forests and have narrow distributions making them prone to extinction. Following a drastic decline in primary forest cover over the last century, many species have not been seen for decades, leading Koopowitz et al. (1998) and Sohmer (2001) to assume that more than half of the Philippine species of *Psychotria* are extinct. In contrast, Sohmer and Davis (2007) provided a more optimistic estimate of extinction rates of up to 28%, but a few of these presumably extinct species have since been rediscovered by local botanists (Ordas et al. 2019; Biag and Alejandro 2020, 2022; Batuyong et al. 2021). Apparently, several primary forest species can tolerate some disturbance and escape extinction in relatively more widespread secondary forests (Sohmer and Davis 2007; Biag and Alejandro 2022).

## ﻿Taxonomic history of *Psychotriaphilippensis* Cham. & Schltdl.

Five *Psychotria* names could not be assigned in the revision of Sohmer and Davis (2007). These include the first *Psychotria* name published for the Philippines and named after the archipelago: *Psychotriaphilippensis* Cham. & Schltdl. (Chamisso and Schlechtendal 1829a). Sohmer and Davis (2007: 238) excluded the name from *Psychotria* but could not assign it to a genus due to the missing type material.

A year after its publication in 1829, *Psychotriaphilippensis* was already in use and was included in the *Prodromus* of de Candolle (1830: 505–506) in an attempt to summarize all known seed plants. However, Candolle did not examine any specimens, and his description is essentially an abbreviated version of that published in the original protologue, a limitation that many subsequent authors had in understanding the identity of the name. Miquel (1857: 282) was the first to conclude that *P.philippensis* was misplaced in *Psychotria* but incorrectly suggested an affinity with *Chassalia*, a palaeotropical genus of the tribe Palicoureeae. Naves and Fernández-Villar (1880: 112, “Verè distinctissima species”) again accepted *P.philippensis* as a species of *Psychotria*, referring to a specimen they identified as this species and had seen in the herbarium of Sebastián Vidal (now at MA, the herbarium of the Real Jardín Botánico, Madrid, Spain). However, the identity of the material they examined seems doubtful since they gave the Caraballo Mountains in central Luzon as the locality, where the presence of the species (a mangrove species, see below) is ecologically impossible. Kuntze (1891) considered *Uragoga* Baill. the correct name for *Psychotria* and proposed the combination *U.philippensis* to accommodate the species. In his enumeration of Philippine Rubiaceae, Elmer (1906: 32) retained *P.philippensis* as an accepted species. Furthermore, he recognized that the elongated, spindle-shaped or fusiform drupes of *P.philippensis* are unusual and differentiate the species from all congeners.

Karl Moritz Schumann (1851–1904), professor of botany and curator at the Berlin herbarium (B), contributed the family Rubiaceae to Engler’s “Die Natürlichen Pflanzenfamilien”. He was the first to place *Psychotriaphilippensis* in the synonymy of the rubiaceous mangrove tree, *Scyphiphorahydrophylacea* (Schumann 1897: 80). He did not give details of his reasoning, but likely he may have seen the type specimen in B, where he worked. Years later, Merrill examined the type at B again (Merrill 1915: 129). He did not reference Schumann’s earlier work but reached the same conclusion and synonymized *P.philippensis* under *S.hydrophylacea* in his “Studies on Philippine Rubiaceae” (Merrill 1915: 129) and in his “Enumeration of Philippine Flowering Plants” (Merrill 1923b: 533).

Most of the Berlin herbarium was destroyed during World War II (Hiepko 1987), the type of *Psychotriaphilippensis* was lost and the identity of the name remained unstudied for several decades. Sohmer and Davis (2007: 238) again excluded the name from *Psychotria* and concluded an affiliation with Rubiaceae but could not assign the name to any genus. The species was not included in the list of Philippine Rubiaceae by Alejandro (2007) but was again listed as an accepted species of *Psychotria* in the checklist of Philippine vascular plants by Pelser et al. (2011 onwards), and subsequently in the World Checklist of Rubiaceae (Govaerts et al. 2022, published online and accessed Oct. 2022, but recently discontinued) and the Plants of the World Online (http://powo.science.kew.org, accessed Feb. 2023), who probably overlooked the synonymizations by Schumann and Merrill or reached a different conclusion on the identity of the name. Wong et al. (2019) gave the most recent treatment and synonymy of *Scyphiphora*, but *P.philippensis* was not listed as a synonym.

The identity of *Psychotriaphilippensis* as a synonym of *Scyphiphorahydrophylacea* is finally established here by a combined analysis of morphological and ecological information in the protologue and an analysis of the historical studies of the name by various authors. Furthermore, the application of the name is fixed by neotypification. This reduces the number of Philippine species of *Psychotria* by one, but fortunately, this is not happening through extinction, as has been the case with too many species of the highly endangered Philippine flora.

### ﻿*Psychotriaphilippensis* and *Scyphiphorahydrophylacea*

Chamisso and Schlechtendal (1829a) described *Psychotriaphilippensis* as a resinous shrub of mangroves (“in maritimis”) with paired axillary or supraxillary inflorescences and subcylindric drupes with acute ridges. Resin production, inflorescence structure and habitat immediately exclude the name from *Psychotria*, which has terminal or solitary-pseudaxillary inflorescences and does not occur in mangroves or other coastal habitats (e.g., Sohmer and Davis 2007; see also Taylor 1996, 2020 and Lachenaud 2019). Instead, the detailed and elaborate description in the protologue is an excellent and unambiguous match for *Scyphiphorahydrophylacea*, a rare and endangered mangrove species that is distributed from East India and Madagascar to the Western Pacific and Northern Australia, including the Philippines (e.g., Puff and Rohrhofer 1993; Alejandro and Liede 2003; Alejandro 2007; Almazol and Cervancia 2013). Thus, there is no reason to doubt the identifications of Schumann (1897: 80) and Merrill (1915, 1923b), who did extensive work on Philippine and other Rubiaceae and were probably the only botanists to examine any of the type specimens.

The monotypic *Scyphiphora* is unusual within Rubiaceae in a number of ecological and morphological characters and is surveyed in detail by Puff and Rohrhofer (1993): It is the only member of the family that occurs in mangrove vegetation. Thus, it is exposed to the influence of salt or brackish water, which only a few Rubiaceae, such as the coastal *Guettardaspeciosa* L. (Guettardeae) and *Hydrophylaxmaritima* L. f., can withstand (e.g., Puff et al. 2021). *Scyphiphora* is typically associated with species of the Rhizophoraceae and other mangroves but is never a dominant component. Its phylogenetic position was long unclear until molecular phylogenetic data showed that it belongs to the Vanguerieae alliance within the subfamily Ixoroideae and is best placed in the monotypic tribe Scyphiphoreae Kainul. & B. Bremer (Mouly et al. 2009; Razafimandimbison et al. 2011; Kainulainen et al. 2013). Finally, recent phylogenetic studies have demonstrated that Scyphiphoreae is sister to the monotypic Southwest Chinese Trailliaedoxeae Kainul. & B. Bremer, and together are sister to the *Glionnetia*–Vanguerieae–Greeneeae–Ixoreae clade (Wikström et al. 2020).

The fruits of *Scyphiphora* are somewhat reminiscent of those of *Psychotria*. Both genera have bilocular drupaceous fruits, but *Psychotria* has fleshy, usually red, bird-dispersed drupes, usually with two one-seeded pyrenes embedded in the fruit pulp. When fresh, these fruits are more or less globose to ovoid or obovoid, and the longitudinally ribbed pyrene surface becomes apparent only in dried fruits (e.g., Taylor 1996, 2020; Sohmer and Davis 2007; Lachenaud 2019). The only exceptions from that fruit type are species of the former genus *Cremocarpon* and *Pyragra* with dry schizocarps nested within a broadly defined *Psychotria* (e.g., Razafimandimbison et al. 2014; Taylor 2020).

Meanwhile, the fruits of *Scyphiphora* are drift fruits adapted for sea dispersal, a rare dispersal mechanism in the family, otherwise found in few other members of the family that occur in coastal vegetation, for example, in *Guettardaspeciosa*. The fruits of *Scyphiphora* are two-locular with two superposed ovules per locule, one ascending, the other descending. When the fruits are mature, they are elongated, dry, brownish and strongly longitudinally ridged. Most of the pericarp and the prominent ribs are composed of mesocarp, which consists of dead, thin-walled, lignified cells. Their lumina are connected by numerous pits and are probably air-filled to increase their buoyancy. The skin-like exocarp is thin, parenchymatic, dry and usually detaches to some extent when the fruits mature. Finally, the endocarp is thin, dry, and heavily sclerified, enclosing each of the two locules. In mature fruits, the seeds are connate by layers of parenchymatic cells, the fruits therefore do not separate into mericarps and the seeds are dispersed together. As pointed out by Puff and Rohrhofer (1993), the fruits were often referred to as “fleshy” or “drupes”, but the entire pericarp is dry when mature and these terms therefore are inaccurate.

### ﻿Chamisso and his collections

Between August 1815 and August 1818, the Chancellor of the Russian Empire, Count Nikolai Romanzoff (1754–1826), commissioned an expedition around the world on the Russian brig Rurik under the command of Captain Otto von Kotzebue (1787–1846). In addition to their primary goal of finding the Northeast Passage from the Bering Strait to the Atlantic Ocean, they aimed to make scientific collections, for which they hired the famous poet and belated botanist Ludolf Karl Adelbert von Chamisso (1781–1838; see Schlechtendal 1839 and Schmid 1942 for biography and bibliography), zoologist Johann Friedrich von Eschscholtz (1793–1831) and artist Louis Choris (1795–1828). The extensive diaries and travelogues of Chamisso (1836a, 1836b), Kotzebue (1821) and Choris (1822) give rather detailed contemporary views of the Romanzoffian expedition, and Bździach (2004) and Maaß (2016) provided excellent historical contextualization.

In his first report to Romanzoff, Chamisso (1818: 206) estimated that they had collected about 2,500 plant species, one-third of which were undescribed. Upon returning from the expedition, Chamisso received permission to take his entire collection to Berlin to study and publish the taxonomic novelties, where they remained until his death (Hiepko 2004; Maaß 2016: 134; see also Chamisso 1818: 208). To this day, the total size of the collections remains unknown. Still, it is clear that the same species were often collected in several places, and each gathering consisted of numerous duplicates, which partly explains the wide dissemination of many Chamisso specimens (e.g., Maaß 2016: 171–172).

Chamisso donated a complete set of specimens from the expedition on the Rurik to the Berlin Herbarium (B). As stipulated in his will, an additional set of “1,800 plant species” was given to his successor at B, Johann Friedrich Klotzsch, who also donated them to the herbarium (Schlechtendal 1839: 104; Urban 1917: 19, 22, 336). Unfortunately, most of B was destroyed during World War II, including most of the two sets of Chamisso’s collections deposited there (Hiepko 1987). Duplicates are expected in HAL (see below), where the herbarium of his close friend Schlechtendal, professor of botany and director of the Botanical Garden of the University of Halle, is kept (Werner 1988; Braun and Wittig 2003). Schlechtendal received part of Chamisso’s collections during their joint work in Berlin on the publication of the plants from the expedition on the Rurik (e.g., Chamisso 1826, 1830; Chamisso and Schlechtendal 1826a, b, 1827, 1829a, b).

In 1840, two years after his death, Chamisso’s private herbarium, which contained up to 12,000 species and 60,000 specimens of his and many of his contemporary collectors (Ruprecht 1864: 4), was acquired by the St. Petersburg Academy of Sciences and is now in the herbarium of the Komarov Botanical Institute (LE). This collection is said to include most of the specimens from the Romanzoffian expedition, but the specimens are generally unmounted and difficult to access and study (Imchanitzkaja 2004).

## ﻿Taxonomic treatment

### ﻿ Scyphiphora hydrophylacea

Taxon classificationPlantaeGentianalesRubiaceae

C.F.Gaertn., Suppl. Carp. 1(2): 91–92, tab. 196, fig. 2. 1806.

F9D4920E-D493-5A43-AED5-C4B2F625BBFC

= Epithiniamalayana Jack, Malayan Misc. 1(5): 12–13. 1820. ≡ Scyphiphoramalayana (Jack) Bedd., Fl. Sylv. S. India Forester’s Man. Bot.: cxxxiv–3, tab. 29., fig. 5. 1874, nom. inval. Type: Singapore. Singapore Island [protologue: “Found in Mangrove swamps on the Island of Singapore.”], s.d., *W. Jack s.n.* (lectotype, designated by Wong et al. 2019: 283: L [L 0001344]).
= Psychotriaphilippensis Cham. & Schltdl., Linnaea 4(1): 21–22. 1829a. ≡ Uragogaphilippensis (Cham. & Schltdl.) Kuntze, Revis. Gen. Pl. 2: 962. 1891. Type: Philippines. Luzon, Calabarzon Region, Cavite Province: Noveleta [protologue: “Legimus in maritimis circa Tierra-alta Luçoniae.”], 1817–1818, *L. K. A. von Chamisso s.n.* (type, B†). Neotype: Philippines, Palawan Province: Culion Island, August 1913, *L. Escritor s.n.* in *Merrill: Species Blancoanae 635* (neotype, here designated: US [00624079]; isoneotypes: L [L.2962064], P [P03972577], W [W 0131765]).
= Ixoramanila Blanco, Fl. Filip.: 60–61. 1837. Type: Philippines. Palawan Province: Culion Island, August 1913, *L. Escritor s.n.* in *Merrill: Species Blancoanae 635* (first-step neotype, designated by Merrill 1918: 364; second-step neotype, here designated: US [00624079]; isoneotypes: L [L.2962064], P [P03972577], W [W 0131765]).
= Hydnophytumcostatum Drake, J. Bot. (Morot) 9: 240–241. 1895. Type: Vietnam. Quảng Ninh Province: Surroundings of Quảng Yên [protologue: “Environs de Quang-Yen, au milieu des palétuviers (685).”], August 1885, *B. Balansa 685* (lectotype, designated by Wong et al. 2019: 283: P [P00836559]).

#### Type.

Indonesia. Java, Jawa Barat: Anyer [protologue: “*Hydrophylax*. Collect. Banks.”], 2. Oct. 1770, *unknown collector* in *J. Banks s.n.* (lectotype, here designated: BM [BM000945301]).

##### ﻿The type of *Psychotriaphilippensis*

*Psychotriaphilippensis* was published in a series on the botanical results of the Romanzoffian expedition on the Rurik prepared by Chamisso and Schlechtendal (1829a). The protologue gives “in maritimis circa Tierra-alta Luçoniae” as a rather minimalistic collection locality. Fortunately, more detailed information can be derived from the diaries and travelogues of Chamisso (1836a, b) and Kotzebue (1821). According to them, the Rurik stayed in the port of Cavite from 18 December 1817 to 28 January 1818 for refurbishments, the most important Philippine fortress and an arsenal of the Spaniards at that time. Knowing that Cavite had little to offer a botanist, Chamisso quickly settled in Tierra Alta, then a small village on the high shore of Manila Bay, where the sandy headland of Cavite joins the adjacent mainland.

Chamisso noted that the lush forests around Tierra Alta extend from the mountains to the coast, where “*Rhizophora* and other trees reach into the sea” (Chamisso 1836b: 118). He spent most of his time in the Philippines here and roamed the area around Tierra Alta, where he also collected the type specimen of *Psychotriaphilippensis* (Chamisso and Schlechtendal 1829a). Chamisso first passed through Tierra Alta on 27 December 1817, when he travelled overland from Manila back to Cavite. The French nobleman Don San Jago de Echaparre offered him hospitality there, and he returned to work in Tierra Alta a few days later. He stayed there until 12 January 1818, when he first left for an eight-day expedition to Taal Lake and Volcano in the interior, returning to Cavite shortly thereafter.

The type collection of *Psychotriaphilippensis* (apparently mentioned by him as one of the “other trees” in the remark quoted above) can thus be dated and localized to the mangroves around Tierra Alta and a period of about two weeks. According to information on the neotype, US 00624079, *Scyphiphorahydrophylacea* was already extinct in the entire Manila Bay region by around 1913, where it was once widespread (Blanco 1837: 60–61, as *Ixoramanila*). This is even more tragic as Blanco reported that the native Tagalog names he attributed to the species, *nilad* and *manilad* or *may-nilad*, places where *nilad* is abundant, ultimately led to the name Manila. This notion is, however, rejected by modern authors (e.g., Baumgartner 1975).

After the destruction of B, where Schumann and Merrill had seen the only known type specimen, there should have been additional original material of *Psychotriaphilippensis* found in HAL and LE, which hold the largest extant parts of Chamisso’s herbarium (see above). However, no specimens are currently known in either of these herbaria (HAL: Braun and Wittig 2003; LE: Larisa Orlova, personal comment) or other collections (JSTOR Global Plants database, http://plants.jstor.org; accessed February 2023). Searches in the herbarium of the Natural History Museum, Vienna (W), where three Chamisso specimens of *Psychotria* recently resurfaced in the extensive private herbarium of Stephan Ladislaus Endlicher (1804–1849), professor of botany and director of the Botanical Garden and Botanical Museum of Vienna, were also unsuccessful (Berger 2018; see also Bräuchler et al. 2021).

Therefore, the name *Psychotriaphilippensis* is neotypified here, fixing the application of the name after nearly two centuries of uncertainty (ICN, Art. 9.8, 9.13; Turland et al. 2018). The specimen designated as neotype was collected by Leonicia Escritor on Culion Island, Palawan, and is no. 635 of the exsiccatae series “Species Blancoanae” issued by Merrill. In this series, he distributed selected specimens that he considered particularly characteristic of the species published by Francisco Manuel Blanco (1778–1845) in the first two editions of his “Flora de Filipinas” (Blanco 1837, 1845) and the third edition edited by Andrés Náves (Blanco 1877). Blanco did not preserve specimens, and the interpretation of some of his names remained problematic until Merrill (1918) published his critical revision “Species Blancoanae” after a thorough study of the publication. In 1916, he issued a corresponding set of “illustrative specimens” for each of Blanco’s names as he understood them.

For example, Merrill (1918: 364) identified *Ixoramanila* (Blanco 1837) as the previously published *Scyphiphorahydrophylacea* and distributed the specimens mentioned above to clarify the application of the name *Ixoramanila* (see also Naves and Fernández-Villar 1880: 109, tab. 277). Thus, he anticipated the concept of the neotype, and his “illustrative specimens” can be understood today as first-step neotypifications of the respective names. In a subsequent second-step neotypification the designation may be narrowed to a single specimen (ICN, Art. 9.17, Turland et al. 2018; see also Nicolson and Arculus 2001). Indeed, many of Blanco’s names were later neotypified with specimens from Merrill’s “Species Blancoanae” (e.g., Nicolson and Arculus 2001).

According to a letter preserved at the United States National Herbarium (US), Merrill prepared 15 sets of his “Species Blancoanae”, each with 1046 specimens, and sent the first to US. Furthermore, he stated that the set at US was the only set that included original data such as field labels or notes and typewritten drafts of the treatments in the “Species Blancoanae”. According to Nicolson and Arculus (2001), specimens from these sets are now represented in many herbaria including A, B, BM, BO, CAL, F, GH, K, L, MO, NSW, NY, P, U, UC, US and W. These usually have minimal labels stenciled “Merrill: Species Blancoanae No.” in black ink followed by a number stamped in blue ink (Nicolson and Arculus 2001).

Although the above-mentioned gathering “Species Blancoanae” 635 is not from the Manila Bay, it serves as an excellent neotype for *Psychotriaphilippensis* because the gathering has duplicates in many herbaria and agrees with both Merrill’s concept of *Scyphiphorahydrophylacea* and his interpretation of the type of *P.philippensis* as its synonym. Therefore, a specimen of the gathering is here designated as the neotype of *P.philippensis* and the second-step neotype of *Ixoramanila*.

##### ﻿The type of *Scyphiphorahydrophylacea*

With the application of *Psychotriaphilippensis* fixed, some further notes on *Scyphiphora* and *Scyphiphorahydrophylacea* seem useful. As to the type of genus and species, the names were published by the German botanist Carl Friedrich von Gaertner (1772–1850, original German orthography Karl Friedrich von Gärtner) in a “Supplementum” (Gaertner 1806) to his father Joseph Gaertner’s (1732–1791, original German orthography Joseph Gärtner) pioneering work on fruit and seed morphology “De fructibus et seminibus plantarum” (Gaertner 1788, 1790–1792). In writing his “Supplement”, C. F. Gaertner made extensive use of his father’s fruit and seed collection, which was largely based on specimens received from contemporary botanists. One of the most important sources J. Gaertner consulted was Banks’s herbarium in London in 1778, and large parts of his ‘Carpologia’ were based on material that he received on loan or as duplicates through Banks’ generosity (Deleuze 1805: 23–24; Stafleu 1969). Following in his father’s footsteps, C. F. Gaertner also travelled to London in 1802 with the same aims (Stafleu 1969).

J. Gaertner’s collection of fruits and seeds is kept in the herbarium of the University of Tübingen (TUB), as is the herbarium of his son, who added to his father’s carpological collection and kept his own herbarium (Stafleu 1969). Yet, there is no extant original material of *Scyphiphorahydrophylacea* in the Gaertner herbarium at TUB (Uta Grünert, personal comment). However, the protologue (“*Hydrophylax*. Collect. Banksian.”) indeed gives reference to traveler, naturalist and patron of science, Sir Joseph Banks (1743–1820), whose extensive private herbarium later became the foundation of the herbarium at the Natural History Museum, London (BM). There are a number of specimens and drawings of *S.hydrophylacea* at BM associated with Banks, his herbarium and endeavors.

Two specimens, both mounted together on one sheet, are curated there as types of the name. The sheet is annotated in pencil as type of *Scyphiphorahydrophylacea* with the place of publication of the name. The two specimens are labelled ‘1’ and ‘2’ in black ink on the mounting tape at the base of the shoots. The numbers correspond to meagre annotations of locality and collector on the verso of the sheet in the upper left corner, written in black ink, as was common practice in many herbaria at that time. These annotations are here-interpreted as being in the hand of Samuel Törner, who was employed as amanuensis in the Banks herbarium from 1792 to 1797 (Marshall 1978).

Specimen ‘1’, BM 000945301, mounted on the upper left side of the sheet and annotated on the verso ‘1. Java prope Angerpoint. J. B.’, is a small fragment of a fruiting branch with a single leaf. ‘J. B.’ refers to Joseph Banks and links the specimen to James Cook’s first voyage aboard HMS Endeavour from 1768 to 1771, which is confirmed by a printed label of a later date reading ‘Java 1770–71 Banks & Solander’. The specimen was collected at ‘Angerpoint’, here identified as the present-day coastal town of Anjer or Anyer on Java, a natural harbor and important victualling station during the passage of the Sunda Strait. The diaries and travelogues of Cook and Sydney Parkinson (1745–1771), Banks’ illustrator (Hawkesworth et al. 1773: 705; Parkinson 1773: 171; Cook 1893: 349), reported that a boat was sent ashore at Anger Point on 2 October 1770, to gather supplies, and the specimen was most likely collected then. However, neither Banks nor botanist Daniel Carlsson Solander (1733–1782) went ashore that day, as can be seen from Banks’s travelogue published by Hooker (1896: 363). For that reason, the gathering must have been made by one of the sailors gathering supplies, which probably explains the scrappy condition of the specimen.

Specimen ‘2’, BM 000945302, consists of four flowering branches and is annotated ‘2. Paolo Candor. Dav. Nelson’. It was collected by David Nelson (1740–1789), who participated in Cook’s third voyage aboard HMS Resolution (1776–1780) on behalf of Banks. The modern transcription of ‘Paolo Candor’ is Pulo Condor, now Côn Sơn Island, Vietnam. The gathering can be dated to 21–28 January 1780, based on King’s (1784: 450–464) account of the Resolution’s visit to the island.

Banks and his collaborators had already collected *Scyphiphorahydrophylacea* in flower and fruit in Australia, which the Endeavour expedition visited before returning home via the Dutch East Indies and the Cape of Good Hope (Banks et al. 1901: 46). Based on these gatherings, illustrations were produced under the unpublished name ‘Ixoroides littoralis’, and the corresponding artwork is held in the “Cook First Voyage Artwork Collection” in the library of the Natural History Museum in London. These materials include a first pencil sketch with color notes by Parkinson, made in 1770 somewhere at the mouth of the Endeavour River (plate number A4/169A). After Parkinson died on the return from the voyage in 1771 (see Parkinson 1773), the watercolors were completed by James Miller (plate number A4/169B, A4/169 2) and engraved by Charles White (plate number A4/169C, also water colored A4/169 5), both under the patronage of Banks. The illustration remained unpublished for over a century until a posthumous publication on Banks’s Australian plants from the expedition, edited by James Britten (Banks et al. 1901: 46, tab. 143). Corresponding specimens that could be located are BRI-AQ0450760, P00836560 and W0131767, all received from BM, and the former two are curated as types of *S.hydrophylacea*.

Another notable holding of *Scyphiphorahydrophylacea* material at BM is a long-unpublished watercolor by Ferdinand Bauer, made during the 1801–1803 circumnavigation of Australia on HMS Investigator under Captain Matthew Flinders. The watercolor shows a complete specimen with analysis of flowers and fruits, and was based on gatherings from the Northern Territory, also in the library of the Natural History Museum in London (Botany Library no. 70, Admiralty Library no. 122; see Mabberley and Moore 1999: 122, plate 70).

The materials of the Investigator expedition (1801–1803) were definitely not available at the Banksian herbarium during C. F. Gaertner’s visit in 1802. It is also unlikely that the Australian materials of the Endeavour expedition (1768–1771) were seen by him, although they were surely accessioned in a timely manner after Banks’ return. The specimens were gathered under the herbarium name ‘Ixoroides littoralis’, and there is no reference to that name in the protologue of *Scyphiphorahydrophylacea*, where the material seen by Gaertner in the Banks herbarium is instead referred to the genus *Hydrophylax* L. f. (Rubiaceae: Spermacoceae). The only species currently assigned to that genus, *Hydrophylaxmaritima*, inhabits sea shores in India, Sri Lanka, Bangladesh and the western Thai part of the Malay peninsula. It has indehiscent fruits that superficially resemble *Scyphiphora* (e.g. Groeninckx et al. 2009; Puff 1986; Puff et al. 2021), which explains the use of the generic name for the undescribed material in the Banksian Herbarium, and also lends its name to the species epithet. Hence, the two specimens curated as types of *Scyphiphora* at BM remain to be considered as original material.

C. F. Gaertner described and illustrated the fruits of *Scyphiphorahydrophylacea*, but made no mention of the flowers (Gaertner 1806: “Cor. … Stam. …”) and the vegetative parts as for many other species that he described. Therefore it seems more likely that C. F. Gaertner either received isolated fruits on loan and did not study one or both of the complete specimens, or that the material had already been studied or received on loan by J. Gaertner during his visit in 1778, two years before the collection of Nelson’s specimen, and that C. F. Gaertner built upon sketches, manuscripts or other materials inherited from his father. For these reasons, the flowering specimen gathered by Nelson (BM000945302) is not considered original material here, and the single fruiting specimen, BM000945301, is here designated as lectotype of the name *S.hydrophylacea*.

A few years after Gaertner’s (1806) publication, the plants were again described as a monotypic genus, *Epithinia*, by Jack, based on one of his gatherings from Singapore (Jack 1820: 12–13). *Epithiniamalayana* was first synonymized with *Scyphiphorahydrophylacea* by Korthals (1851: 203–204), and from Miquel (1857: 238–239) and especially Gray (1859: 307), onwards the names were consistently considered synonyms. The combination *S.malayana* (Jack) Bedd. was ascribed to Beddome and considered a valid name in various publications and databases. Although the name indeed first appears in the caption accompanying the respective figure analysis in his rare *Foresters Manual* (Beddome 1874: tab. 29, fig. 5), it is not accepted in the corresponding text (Beddome 1874: cxxxiv–3, using a combination of Roman and Arabic pagination). There, Beddome explains that the name used in the figure should be changed to *S.hydrophylacea* (“Under the name of Scyph. malayana, which should be altered as above.”). Apparently, the plates were engraved with the name before the text was finished, and Beddome changed his mind on the identity of the material during the completion of the text, as with few other species in the text. *S.malayana* is therefore not accepted by Beddome and is not valid according to Art. 36.1 (ICN, Turland et al. 2018). Wong et al. (2019: 283) designated the only currently known duplicate of the respective gathering *W. Jack s.n.* at L as lectotype.

Finally, the plants were again described as *Hydnophytumcostatum* Drake (Drake del Castillo 1895) and based on *B. Balansa 685* gathered in Vietnam. Again, the single known specimen was designated as lectotype by Wong et al. (2019: 283).

##### ﻿Bibliography of *Scyphiphora*

Selected and more or less useful historic and contemporary information and illustrations on *Scyphiphorahydrophylacea* can be found in chronological order in the following publications, some of them under synonyms added in parentheses:

Gaertner (1806: 91–92, tab. 196, fig. 2), Jack (1820: 12–13, as *Epithiniamalayana*), Blume (1826–1827: 955), Chamisso and Schlechtendal (1829a: 21–22, as *Psychotriaphilippensis*), de Candolle (1830: 477–478, as *E.malayana*, 505–506, as *P.philippensis*, 577), Richard (1830: 79, tab. 4, fig. 1), Wight and Arnott (1834: 423–424, as *E.malayana*), Blanco (1837: 60–61, as *Ixoramanila*), Endlicher (1838: 525–526, 545, on the latter page as *E.malayana*), Korthals (1851: 203–204), Griffith (1854a, b: 269–271, tab. 478, as *E.malayana*; 1854b: tab. 644A, is the same figure in different composition but named *Lumnitzerapentandra*, the corresponding text accompanying the figure – 1854a: 684 – apparently describes the actual *Lumnitzera*), Hasskarl (1855: 16–17), Miquel (1857: 238–239), Gray (1859: 307), Bentham and Mueller (1867: 417–418), Beddome (1874: 134-3, tab. 29, fig. 5), Kurz (1877: 4), Hooker (1880: 125), Naves and Fernández-Villar (1880: 109, tab. 277), Vidal (1883a, b: 29, tab. 57, fig. G, 1886: 154), Trimen (1894: 337), Schumann (1897: 80, fig. 29 B, C), Banks et al. (1901: 46, tab. 143), Koorders and Valeton (1902: 124–127), King and Gamble (1904: 227–228), Elmer (1906: 32), Koorders (1912: 258), Koorders and Valeton (1915: fig. 563), Merrill (1918: 364, 1923b: 533), Pitard (1922–1924: 280–282, tab. 23, figs 5–8), Ridley (1923: 88–89), Watson (1928: 84–87, tab. 43), White (1929), Valeton (1930: 303), Corner (1940: 559), Henderson (1950: 217–218, fig. 196), Bakhuizen van den Brink (1955: 101–102, 1975: 34–35), Backer and Bakhuizen van den Brink (1965: 316), Wong (1988: 197), Wong (1989: 408), Keng (1990: 161), Hộ (1993: 206, fig. 7607), Puff and Rohrhofer (1993, richly illustrated), Turner (1995: 446), Ridsdale (1998: 235), Mabberley and Moore (1999: 122, pl. 70), Banerjee et al. (2002: 262), Puff et al. (2005: 82, pl. 3.1.19), Tao et al. (2011: 323) and Wong et al. (2019: 282–285, fig. 71).

## Supplementary Material

XML Treatment for Scyphiphora hydrophylacea
